# Accuracy and reliability of imaging modalities for studying bipolar bone loss in anterior shoulder instability: A systematic review

**DOI:** 10.1002/ksa.12531

**Published:** 2024-11-04

**Authors:** Marco Adriani, Maristella Francesca Saccomanno, Andrea Bergomi, Francesco De Filippo, Valerio Daffara, Giuseppe Milano

**Affiliations:** ^1^ Department of Medical and Surgical Specialties, Radiological Sciences, and Public Health University of Brescia Brescia Italy; ^2^ Department of Bone and Joint Surgery Spedali Civili Brescia Italy

**Keywords:** accuracy, anterior shoulder instability, bone loss, glenoid track, imaging modalities, reliability

## Abstract

**Purpose:**

Recurrent shoulder instability, a common musculoskeletal disorder, often involves glenoid bone loss and Hill–Sachs lesions. However, the optimal imaging modality for accurately and reliably quantifying bipolar bone loss remains uncertain. This systematic review aims to evaluate the accuracy and reliability of various imaging modalities in assessing bipolar bone loss in anterior shoulder instability.

**Methods:**

Major electronic databases were searched for English‐language studies reporting the measurement of glenoid track width and/or determination of on/off track HSL through imaging. Studies reporting statistical measures such as area under the curve, sensitivity, specificity, positive predictive value, NPV, intraobserver reliability and interobserver reliability were included. Data extraction and risk of bias assessment were performed independently by two reviewers.

**Results:**

The systematic review included 19 studies comprising 1567 shoulders. Overall, studies could be divided into those looking at the accuracy or reliability of determining glenoid track width, on‐ or off‐track Hill–Sachs lesions and near‐track lesions. Three‐dimensional images of computed tomography (3D‐CT) was the most reliable and accurate imaging modality to measure the glenoid track width. On the opposite, two‐dimensional magnetic resonance imaging (2D‐MRI) did not provide enough evidence of accuracy and reliability in the determination of On/Off track lesions and near‐track lesions.

**Conclusion:**

3D‐CT demonstrated excellent reliability for measuring glenoid track width. However, the reliability of 2D‐MRI for determining on/off track Hill–Sachs lesions is still controversial.

**Level of Evidence:**

Level III.

**Abbreviations**
3D‐CT: three‐dimensional images of computed tomography
ABR: arthroscopic Bankart repair
AUC: area under the curve
BBD: bipolar bone defects
DA: diagnostic accuracy
DAS: diagnostic accuracy studies
DTD: distance to dislocation
GBD: glenoid bone defects
GT: glenoid track
H/G: Hill–Sachs interval to glenoid track width
HSL: Hill–Sachs lesions
ICC: intraclass correlation coefficient
INTER: interobserver reliability
INTRA: intraobserver reliability
k: kappa statistics
MRI: magnetic resonance imaging
NPV: negative predictive value
PPV: positive predictive value
SENS: sensitivity
SPEC: specificity

## INTRODUCTION

Recurrent anterior shoulder instability occurs in up to 60% of patients who experienced acute traumatic dislocation episodes [17]. Osseous injuries on both the glenoid and the humerus have been identified in the last decade as crucial factors in the risk of recurrence and the decision‐making process for the treatment of recurrent anterior shoulder instability [19]. Bipolar bone defects (BBDs) have been reported to be present in 47.4%–70.6% of shoulders one year after the first‐time dislocation [31]. However, the relationship between the glenoid bone defect (GBD) and the Hill–Sachs lesion (HSL) is still a matter of debate. Yamamoto et al. first introduced the concept of the ‘glenoid track’ (GT) to describe the glenohumeral contact areas across the range of motion in normal shoulders [39]. Subsequently, Di Giacomo et al. analyzed kinematic models of the unstable shoulder according to the GT concept and identified two distinct patterns of BBD, named ‘on‐track’ and ‘off‐track’ lesions, with a low and high risk of redislocation, respectively [9, 16].

Studies have shown that the extent of BBD influences recurrence rates after arthroscopic Bankart repair (ABR), making it essential to assess these defects before and during surgery [14]. The concept of critical and subcritical GBD and HSL as acceptable thresholds of bone deficit with no clinical significance is important for surgical decision‐making and continues to evolve. Recent clinical studies suggested subcritical GBD threshold as low as 17%–13.5% to necessitate additional procedures to the standard ABR [10, 30]. Moreover, some on‐track HSL that have a medial margin in close proximity to the GT, named ‘near‐track’ or ‘peripheral track’ lesions have been proposed to be predictive of poor outcomes following ABR alone [22, 38, 40, 41].

Several imaging modalities are currently available for evaluating the GT and for determining if a BBD is on‐ or off‐track [13]. Current literature indicates that three‐dimensional images of computed tomography (3D‐CT) are the gold standard to estimate BBDs, while magnetic resonance imaging (MRI) is the preferred method for evaluating soft tissue anatomy of the glenohumeral joint [11, 19]. A previous systematic review studied the reliability and accuracy of imaging modalities in assessing HSL morphology, with inconclusive results due to inconsistencies in the literature [35]. However, several authors published recently on diagnostic accuracy (DA) and reliability of imaging modalities in assessing BBDs in recurrent anterior shoulder instability. Therefore, the purpose of our study was to summarize the evidence on the DA and reliability of various imaging modalities in assessing different parameters to estimate BBDs in anterior shoulder instability. Our hypothesis was that 3D‐CT imaging would show the highest DA and reliability.

## METHODS

This systematic review was conducted in accordance with the preferred reporting items for systematic reviews and meta‐analyses statement for reporting systematic reviews and meta‐analyses of studies [27]. The study was preregistered to open Science Framework (https://doi.org/10.17605/OSF.IO/25T78).

### Literature search

Studies were identified by searching major electronic databases from their inception up to 24 June 2024. The search strategy was applied to MEDLINE through the OVID platform (Supporting Information S1: Appendix 1) and adapted for EMBASE. Studies meeting the following criteria were included: English‐language studies reporting the measurement of GT width and/or determination of on/off track BBDs through CT or MRI. Only articles reporting at least one of the following statistical measures of DA and/or reliability were included: area under the curve (AUC), sensitivity (SENS), specificity (SPEC), positive predictive value, negative predictive value (NPV), intraobserver reliability (INTRA) and interobserver reliability (INTER). Studies published not in English language or that involved animals, cadaveric studies or did not include any of the statistical measures listed above were excluded. Two authors independently assessed the eligibility of the screened studies. In cases of disagreement, a consensus was reached through discussion with the senior author.

### Data extraction

The same reviewers independently extracted available data from all eligible studies using a piloted form. Information gathered included the following study characteristics: author and year of publication, number of examiners, imaging modality used for measurements, method of measurements of GT, outcome measured in terms of accuracy and reliability and thresholds adopted.

### Data analysis

According to the most accredited recommendations on measurement properties of reliability [23], the following statistical methods were considered valid for the assessment of INTRA and INTER: kappa (k) statistics for dichotomous/nominal/ordinal scores and intraclass correlation coefficients (ICCs) for continuous scores.

The magnitude of agreement between repeated observations was interpreted according to the values of k coefficients and ICCs as follows: <0 as absent (complete discordance between observations), 0–0.20 as slight agreement, 0.21–0.40 as fair agreement, 0.41–0.60 as moderate agreement, 0.61–0.80 as substantial agreement and 0.81–1 as almost perfect agreement [20].

AUC is an effective way to summarize the overall DA of an imaging modality. It takes values from 0 to 1, where a value of 0 indicates a perfectly inaccurate test and a value of 1 reflects a perfectly accurate test. According to Mandrekar et al. [24], a value of 0.5 suggests no discrimination, 0.7–0.8 is considered acceptable, 0.8–0.9 is considered excellent and more than 0.9 is considered outstanding.

Descriptive statistics were used to summarize findings across the studies.

### Methodological quality

The risk of bias assessment tool used for reliability studies was the Quality Appraisal of Reliability Studies (QAREL) checklist which has been previously tested for validity and has been used in other systematic reviews looking at the reliability of diagnostic tests [23]. The checklist includes 11 evaluation items that assess methodological components separately and are graded as ‘yes’, ‘no’, ‘unclear’ or ‘not applicable’. A high risk of bias was considered if the score was <7, a low risk of bias if ≥8 and a moderate risk of bias if the score was 7.

The risk of bias for diagnostic accuracy studies (DAS) was assessed using the Quality Assessment of Diagnostic Accuracy Studies (QUADAS‐2). The tool consists of four key domains covering patient selection, index test, reference standard and flow of patients through the study [36].

Methodological evaluation was initially conducted independently by two reviewers, and any disagreements were resolved through discussion with the senior author until a unanimous grade was assigned to each item.

## RESULTS

### Literature search

**Figure 1:**
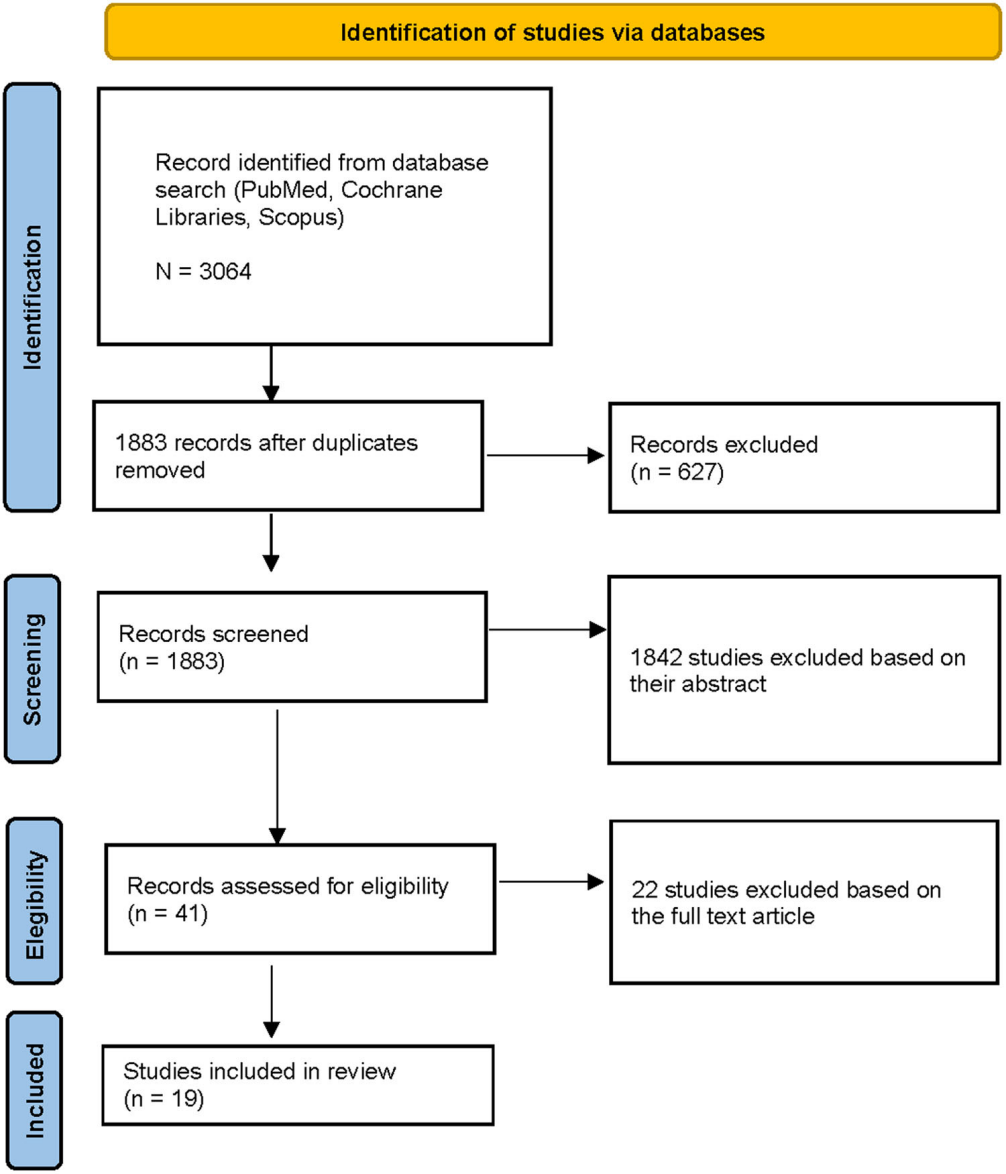
Preferred reporting items for systematic reviews and meta‐analyses flow diagram.

The electronic search resulted in 3064 hits. After removing the duplicates and studies not eligible for inclusion based on their title and abstract, 41 studies remained. Of these, 22 studies were excluded not assessing the eligibility criteria. Nineteen studies were finally included in the review. A diagram of the study selection process can be seen in Figure 1.

### Included studies

Overall, 1567 shoulders were included among the 19 studies [1, 2, 3, 4, 6, 7, 8, 12, 15, 18, 21, 22, 25, 26, 28, 29, 33, 37, 41]. All the included studies reported a preoperative evaluation of the GT with different imaging modalities. The articles were divided into three categories based on the measured outcome. Six of them [2, 8, 15, 18, 25, 37] looked at the reliability in determining the GT width, nine studies [2, 4, 6, 12, 15, 21, 26, 28, 29] reported data on DA or reliability in determining if a BBD is on‐ or off‐track and seven studies [1, 2, 3, 7, 22, 33, 41] evaluated DA and reliability of various imaging modalities in determining near‐track lesions. Single‐study characteristics are reported in detail in Table 1.

**Table 1:** Characteristics of included studies.

References	Shoulders, *n*°	Examiners, *n*°	Imaging modality	Measurement of GT	Data measured	Gold standard for measuring diagnostic accuracy
Barrow 2022 [1]	30	2	2D‐MRI	83% of glenoid width minus GBL	INTRA, INTER, SENS, SPEC, PPV, NPV	Surgical failure
Bhatia 2023 [2]	114	2	2D‐MRI	83% of glenoid width minus GBL	INTER	‐
Boden 2023 [3]	173	2	2D‐MRI	83% of glenoid width minus GBL	INTRA, INTER	‐
Bottoni 2021 [4]	56	3	2D‐MRI	83% of glenoid width minus GBL	INTER	‐
Chalmers 2020 [6]	53	2	2D‐CT; 2D‐MRI	83% of glenoid width minus GBL	INTER	‐
Chen 2021 [7]	222	2	2D‐MRI	83% of glenoid width minus GBL	AUC, SENS, SPEC	Recurrent instability
Di Giacomo 2016 [8]	102	2	3D‐CT	83% of glenoid width minus GBL	INTRA, INTER	‐
Godinho 2021 [12]	44	3	2D‐MRI	83% of glenoid width minus GBL	AUC, SENS, SPEC, NPV, PPV	3D‐CT performed by an experienced orthopaedic
Gyftopoulos 2015 [15]	76	2	2D‐MRI	83% of glenoid width minus GBL	INTRA, INTER, AUC, SENS, SPEC, PPV, NPV	Arthroscopic evaluation before repair
Kawakami 2019 [18]	41	2	3D‐MRI	84% of glenoid width minus GBL	INTRA, INTER	‐
li 2021 [22]	30	2	2D‐MRI	83% of glenoid width minus GBL	INTRA, INTER	‐
Lau 2017 [21]	75	2	2D‐MRI	84% of glenoid width minus GBL	INTRA, INTER	‐
Matsumura 2017 [25]	180	2	3D‐CT	Used 3D reconstructions of CT scans to measure: length = long axis; width = short axis	INTRA, INTER	‐
Metzger 2013 [26]	140	3	2D‐MRI	84% of glenoid width minus GBL	INTER	‐
Schneider 2017 [28]	71	4	3D‐CT	83% of glenoid width minus GBL	INTRA, INTER	‐
Sgroi 2021 [29]	80	2	2D‐CT; 2D‐MRI	83% of glenoid width minus GBL	INTRA, INTER	‐
Verweij 2023 [33]	80	‐	2D‐MRI	83% of glenoid width minus GBL	AUC	Recurrent instability
Wu 2022 [37]	40	2	3D‐CT	83% of glenoid width minus GBL	INTRA, INTER	‐
Yang 18 [41]	160	‐	2D‐MRI	83% of glenoid width minus GBL	AUC, SENS, SPEC	Recurrent instability
**Total**	**1567**					

Abbreviations: AUC, area under the curve; GBL, glenoid bone loss; GT, glenoid track; INTER, interobserver reliability; INTRA, intraobserver reliability; MRI, magnetic resonance imaging; NPV, negative predictive value; PPV, positive predictive value; SENS, sensitivity; SPEC, specificity.

### GT width

All the studies looking at the reliability of various imaging modalities in determining GT width adopted ICC. All of them looked at INTER whereas only five studied INTRA [8, 15, 18, 25, 37]. Overall, three studies determined the GT width using 3D‐CT [8, 25, 37], one used two‐dimensional (2D) MRI [15], one 3D‐MRI [18] and one used both 2D‐CT and 2D‐MRI without distinguishing the reliability of each imaging modality [2]. All studies evaluating 3D imaging (CT or MRI) showed excellent INTRA and INTER. 2D analysis was reported to have good INTRA and INTER in two studies [2, 15]. Reliability coefficient ranges for each imaging modality are reported in Table 2.

**Table 2:** Reliability of determining glenoid track width.

References	INTRA	INTER
3D‐CT		
Di Giacomo 2016 [8]	ICC = 0.916–0.976	ICC = 0.999
Matsumura 2017 [25]	ICC > 0.9	ICC > 0.9
Wu 2022 [37]	ICC = 0.966	ICC = 0.925
2D‐MRI/MRA		
Gyftopoulos 2015 [15]	ICC = 0.73	ICC = 0.85
3D‐MRI		
Kawakami 2019 [18]	ICC = 0.988	ICC = 0.988
MRI and CT		
Bhatia 2023 [2]	‐	ICC = 0.72

Abbreviations: 3D‐CT, three‐dimensional images of computed tomography; CT, computed tomography; ICC, intraclass correlation coefficient; INTER, interobserver reliability; INTRA, intraobserver reliability; MRA, magnetic resonance arthrography; MRI, magnetic resonance imaging.

### On‐/off‐track

All nine studies assessed the reliability of different imaging modalities to estimate BBDs as on‐track or off‐track. Eight of them used k statistics whereas only one preferred using percentage of agreement [28]. All studies evaluated INTER whereas only two evaluated INTRA [21, 28]. 2D‐MRI was the most studied imaging modality with conflicting results as two articles [4, 21] showed excellent agreement whereas the other three [6, 26, 29] reported only moderate k values. Reliability coefficient ranges for each imaging modality are reported in Table 3.

**Table 3:** Accuracy and reliability of determining if an HSL is on‐ or off‐track.

References	AUC	SENS, %	SPEC, %	PPV	NPV	INTRA	INTER
2D‐CT							
Chalmers 2020 [6]	‐	‐	‐	‐	‐	‐	k = 0.62
Sgroi 2021 [29]	‐	‐	‐	‐	‐	‐	k = 0.632
3D‐CT							
Schneider 2017 [28]	‐	‐	‐	‐	‐	%Agr = 80.3‐90.	%Agr = 71.8
2D‐MRI/MRA							
Bottoni 2021 [4]	‐	‐	‐	‐	‐	‐	k = 0.92
Chalmers 2020 [6]	‐	‐	‐	‐	‐	‐	k = 0.57
Godinho 2021 [12]	0.654–0.783	35–65	91.7–95.8	86.7–92.31	63.89–75.86	‐	‐
Gyftopoulos 2015 [15]	0.866	72.2	87.9	65	91	‐	‐
Lau 2017 [21]	‐	‐	‐	‐	‐	k = 0.86	k = 0.81
Metzger 2013 [26]	‐	‐	‐	‐	‐	‐	k = 0.43
Sgroi 2021 [29]	‐	‐	‐	‐	‐	‐	k = 0.413
MRI and CT							
Bhatia 2023 [2]	‐	‐	‐	‐	‐	‐	k = 0.31

Abbreviations: 2D‐CT, two‐dimensional images of computed tomography; AUC, area under the curve; CT, computed tomography; HSL, Hill–Sachs lesion; INTER, interobserver reliability; INTRA, intraobserver reliability; MRA, magnetic resonance arthrography; MRI, magnetic resonance imaging; NPV, negative predictive value; PPV, positive predictive value; SENS, sensitivity; SPEC, specificity.

DA was studied only in two studies [12, 15] and both used 2D‐MRI with conflicting results. Godinho et al. [12] used 3D‐CT as the gold standard and showed a low accuracy among three examiners, whereas Gyftopoulos et al. [15] used arthroscopic evaluation of engagement before repair as the gold standard and revealed excellent DA of 2D‐MRI.

### ‘Near‐track lesions’

All seven studies looking at near‐track lesions adopted 2D‐MRI as the imaging modality. Three of them [2, 7, 41] used the Hill–Sachs interval to glenoid track width (H/G) ratio to determine if an on‐track BBD was near‐track. A cut‐off >0.7 was used in all articles. Only one [2] looked at reliability with only moderate INTER. DA in predicting recurrent instability was considered excellent in both studies evaluating it [7, 41].

Four studies adopted distance to dislocation (DTD) as a measurement to determine if an on‐track BBD was near‐track and so at risk for recurrent instability. Two of them [22, 33] used a cut‐off value ≤ 8 mm whereas Barrow et al. [1] and Boden et al. [3] used 10 mm as threshold. Three studies [1, 3, 22] evaluated reliability and all of them showed excellent INTRA, whereas, INTER ranged from good to excellent. Two studies [1, 33] evaluated DA of DTD in predicting surgical failure or recurrence, with conflicting results. Barrow et al. [1] showed excellent NPV and SPE, whereas Verweij et al. [33] found a shorter DTD to be not accurate in predicting recurrence. Accuracy and reliability coefficient ranges are shown in Table 4.

**Table 4:** Accuracy and reliability of determining if an on‐track HSL is ‘Near‐track’.

References	Imaging modality	AUC	SENS, %	SPEC, %	PPV	NPV	INTRA	INTER
H/G ratio > 0.7							
Bhatia 2023 [2]	2D MRI	‐	‐	‐	‐	‐	‐	K = 0.55
Chen 2021 [7]	2D MRI	0.821	80.6	70.7	‐	‐	‐	‐
Yang 2018 [41]	2D MRI	0.802	71	74	‐	‐	‐	‐
DTD ≤ 8 mm								
Li 2021 [22]	2D MRI	‐	‐	‐	‐	‐	ICC = 0.82	ICC = 0.71
Verweij 2023 [33]	2D MRI	0.49	‐	‐	‐	‐	‐	‐
DTD ≤ 10 mm								
Barrow 2022 [1]	2D MRI	‐	51.7	89	‐	91.8	ICC = 0.95	ICC = 0.96
Boden 2023 [3]	2D MRI	‐	‐	‐	‐	‐	ICC = 0.82	ICC = 0.71

Abbreviations: AUC, area under the curve; H/G, Hill–Sachs interval to glenoid track width; HSL, Hill–Sachs lesion; ICC, intraclass correlation coefficient; INTER, interobserver reliability; INTRA, intraobserver reliability; MRI, magnetic resonance imaging; NPV, negative predictive value; PPV, positive predictive value; SENS, sensitivity; SPEC, specificity.

### Risk of bias within studies

The risks of bias in the included studies, according to the QAREL checklist and QUADAS‐2 tool are presented in Supporting Information S1: Appendices 2 and 3, respectively. Regarding reliability studies, a total of four studies showed low risk of bias, one had moderate risk, whereas 10 were recognized as at high risk. Common risks of bias were lack of clarity about blinding raters to their own prior findings and blinding to the patient's clinical information and additional cues about participants.

Regarding DASs, methods for participant selection had a high risk of bias in two out of six studies (33%) while regarding the flow of patients, five studies (83%) were found to be at high risk of bias due to the nonapplication of the reference standard to all patients.

## DISCUSSION

The main finding of this review is that GT width can be reliably measured using both 2D‐MRI and 3D‐CT scans, whereas there is still not enough evidence in the literature to consider 2D‐MRI reliable in determining if a BBD is on‐ or off‐track.

Particularly, GT width measurements on 3D‐CT scans showed excellent INTRA and INTER, whereas 2D‐magnetic resonance arthrography (MRA) had good INTRA and excellent INTER. No studies reported on 2D‐CT scans and, therefore, no conclusions can be made. Moreover, only one study analyzed the reliability of 3D‐MRI in measuring GT width, and, therefore, more studies are needed in order to come to safe conclusions.

Stillwater et al. [32] compared the accuracy of 3D‐MRI and 3D‐CT in measuring GBD and HSL and showed that the two modalities have similar accuracy. The primary advantage of 3D‐MRI is related to radiation avoidance. However, it must be highlighted that 3D MRI is still not a popular feature by using common DICOM viewer software, therefore, 3D‐CT scan probably remains the easiest option.

Although 2D‐MRI/MRA was the most studied imaging modality in determining if a BBD is on‐ or off‐track, reliability analysis showed conflicting results. Three articles reported only moderate INTER, whereas the other two studies reported excellent INTER, with kappa coefficient values ranging between 0.41 and 0.92 [4, 6, 21, 26, 29]. This is in contrast with a previous systematic review that reported sufficient evidence to support the use of MRI imaging modality for assessing the GT [34]. This could be due to the fact the only recent papers questioned previously acquired reliability data [6, 29]. Additionally, two recent studies compared 2D‐MRI and CT scans [6, 12]. Chalmers et al. [6] showed how MRI tends to underestimate both GBD and combined loss, whereas Godinho et al. [12] found that 2D‐MRI/MRA tends to yield lower Hill–Sachs Interval values when compared to 3D‐CT.

New thresholds are emerging to discriminate on‐track lesions at risk of surgical failure after Bankart repair. Yang et al. introduced the concept of H/G ratio and Li et al. introduced the DTD [22, 41]. These measurements help to identify HSL with the medial margin of the lesion in close proximity to the GT. Those lesions have been defined as ‘near‐track’ [7] or ‘peripheral track’ [38, 40]. Interestingly, all the papers included used 2D‐MRI to measure DTD or H/G ratio. As previously stated, the use of MRI may underestimate the amount of true bone loss if a suboptimal sagittal slice is used [6]. Li et al. [22] and Barrow et al. [1] are the only two articles reporting this as a limitation of their studies. Unfortunately, there were not enough studies to make a comparison between DTD and H/G ratio in terms of reliability and accuracy. Reliability was considered excellent for DTD in all the articles reporting data on it, whereas the H/G ratio was shown to have only moderate INTER in one single study. DA in predicting recurrent instability was evaluated in three articles [7, 33, 41]. Two of them used the H/G ratio to determine near‐track lesions, with a threshold of 0.7 [7, 41]. AUC ranged from 0.802 to 0.821 with a SENS of 71%–80.6% and a SPEC of 74%–70.7%. The high DA observed underscores the importance of evaluating this new GT concept, which considers both off‐track lesions and near‐track or peripheral on‐track lesions at higher risk of surgical failure. On the other hand, Verweij et al. [33] looked at the ability of shorter DTD to predict recurrence after ABR showing very low accuracy. However, they included only a military population, and their analysis was underpowered. It has previously been shown that the effect of the GT concept seems to be smaller in a military population and, therefore, a larger sample size is needed to identify the effect [5].

The present review is the first to summarize data on the accuracy and reliability of GT width and near‐track measurements with different imaging modalities. The clinical relevance is that caution is advised when evaluating BBDs with 2D‐MRI, given the limited evidence regarding its reliability. To date, 3D‐CT should be considered the most reliable imaging modality for GT assessment.

The limitations of the present study are mainly related to the heterogeneity of the imaging settings that did not allow us to pool a large number of shoulders and perform a meta‐analysis. In addition, only five papers reported on DA of imaging modalities in GT assessment and used different gold standards to compare data. Furthermore, this review included studies with variable levels of evidence and the majority of them had a high risk of bias. Further studies would be necessary that follow the checklist available for reliability and DA studies to decrease methodological biases.

## CONCLUSION

Currently, there is sufficient evidence supporting the use of 3D‐CT for determining GT width. However, the most reliable and accurate imaging modality for assessing on/off track BBDs remains undetermined. Near‐track lesions have garnered increasing attention for their potential role in predicting surgical failure and recurrence, with preliminary evidence supporting the use of 2D‐MRI in their assessment.

## AUTHOR CONTRIBUTIONS

*Conceptualization*: Giuseppe Milano and Maristella Francesca Saccomanno. *Literature search*: Andrea Bergomi, Francesco De Filippo and Valerio Daffara. *Data analysis*: Marco Adriani, Andrea Bergomi, Francesco De Filippo and Valerio Daffara. *Writing—original draft preparation*: Marco Adriani. *Writing—review and editing*: Giuseppe Milano and Maristella Francesca Saccomanno. *Supervision*: Giuseppe Milano.

## CONFLICT OF INTEREST STATEMENT

The authors declare no conflicts of interest.

## ETHICS STATEMENT

The authors have no funding to report.

## Supporting information

Supporting information.

Supporting information.

Supporting information.

## Data Availability

Data sharing is not applicable to this article as no new data were created or analyzed in this study.
