# Supplementary material for: Accuracy and reliability of imaging modalities for studying bipolar bone loss in anterior shoulder instability: A systematic review
Source: Knee Surg Sports Traumatol Arthrosc. 2024 Nov 4;33(5):1844–52. doi: 10.1002/ksa.12531 (PMC12022830; doi:10.1002/ksa.12531)
Supplement: Supplementary file 1 — Supporting information. [file KSA-33-1844-s003.docx]

**Appendix 1: Search strategy**

1 Imaging

2 Radiographic

3 Computed Tomography

4 Magnetic Resonance Imaging

5 1 or 2 or 3 or 4

6 Hill Sachs

7 Glenoid Bone Loss

8 Humeral Head Bone Loss

9 Shoulder Instability

10 On Track

11 Off Track

12 Glenoid Track

13 Engaging Lesion

14 Non-engaging Lesion

15 6 or 7 or 8 or 9 or 10 or 11 or 12 or 13 or 14

16 5 and 15
