# Supplementary material for: Accuracy and reliability of imaging modalities for studying bipolar bone loss in anterior shoulder instability: A systematic review
Source: Knee Surg Sports Traumatol Arthrosc. 2024 Nov 4;33(5):1844–52. doi: 10.1002/ksa.12531 (PMC12022830; doi:10.1002/ksa.12531)
Supplement: Supplementary file 2 — Supporting information. [file KSA-33-1844-s002.docx]

**Appendix 2:** QAREL checklist

| **Authors** | **1** | **2** | **3** | **4** | **5** | **6** | **7** | **8** | **9** | **10** | **11** | Total score (Yes items) | Overall risk of bias |
| --- | --- | --- | --- | --- | --- | --- | --- | --- | --- | --- | --- | --- | --- |
| Bahtia 2023 | Y | Y | Y | NA | U | Y | Y | NA | Y | Y | Y | 8/11 | LOW |
| Barrow 2022 | Y | Y | U | U | NA | U | U | NA | Y | Y | Y | 5/11 | HIGH |
| Boden 2023 | Y | Y | U | U | NA | U | U | NA | Y | Y | Y | 5/11 | HIGH |
| Bottoni 2021 | Y | Y | Y | NA | NA | U | U | NA | Y | Y | Y | 6/11 | HIGH |
| Chalmers 2020 | Y | Y | Y | NA | NA | U | U | NA | Y | Y | Y | 6/11 | HIGH |
| Di Giacomo 2016 | Y | Y | Y | U | NA | U | U | NA | Y | Y | Y | 6/11 | HIGH |
| Gyftopoulos 2015 | Y | Y | Y | U | NA | Y | Y | NA | Y | Y | Y | 8/11 | LOW |
| Kawakami 2019 | Y | Y | Y | U | NA | U | U | NA | Y | Y | Y | 6/11 | HIGH |
| Lau 2017 | Y | Y | U | U | NA | U | U | NA | Y | Y | Y | 5/11 | HIGH |
| Li 2021 | Y | Y | U | U | NA | U | U | NA | Y | Y | Y | 5/11 | HIGH |
| Matsumura 2017 | Y | U | Y | U | NA | U | U | NA | Y | U | Y | 4/11 | HIGH |
| Metzger 2013 | Y | Y | Y | U | NA | Y | Y | NA | Y | Y | Y | 8/11 | LOW |
| Schneider 2017 | Y | Y | Y | Y | NA | Y | Y | NA | Y | Y | U | 8/11 | LOW |
| Sgroi 2021 | Y | Y | Y | Y | NA | U | U | NA | Y | Y | Y | 7/11 | MODERATE |
| Wu 2022 | Y | Y | Y | U | NA | U | U | NA | Y | Y | Y | 6/11 | HIGH |

| ***Scoring: Y = Yes N = No U = Unclear NA = Not applicable*** | | |  |  |  |  |  |  |  |  |
| --- | --- | --- | --- | --- | --- | --- | --- | --- | --- | --- |
| *Item 1: Was the test evaluated in a sample of subjects who were representative of those to whom the authors intended the results to be applied?* | | | | | | | | | |  |
| *Item 2: Was the test performed by raters who were representative of those to whom the authors intended the results to be applied?* | | | | | | | | |  |  |
| *Item 3: Were raters blinded to the findings of other raters during the study?* | | | |  |  |  |  |  |  |  |
| *Item 4: Were raters blinded to their own prior findings of the test under evaluation?* | | | | |  |  |  |  |  |  |
| *Item 5: Were raters blinded to the results of the reference standard for the target disorder (or variable) being evaluated?* | | | | | | | |  |  |  |
| *Item 6: Were raters blinded to clinical information that was not intended to be provided as part of the testing procedure or study design?* | | | | | | | | |  |  |
| *Item 7: Were raters blinded to additional cues that were not part of the test?* | | | |  |  |  |  |  |  |  |
| *Item 8: Was the order of examination varied?* |  |  |  |  |  |  |  |  |  |  |
| *Item 9: Was the time interval between repeated measurements compatible with the stability (or theoretical stability) of the variable being measured?* | | | | | | | | | | |
| *Item 10: Was the test applied correctly and interpreted appropriately?* | | | |  |  |  |  |  |  |  |
| *Item 11: Were appropriate statistical measures of agreement used?* | | |  |  |  |  |  |  |  |  |
