# Supplementary material for: Accuracy and reliability of imaging modalities for studying bipolar bone loss in anterior shoulder instability: A systematic review
Source: Knee Surg Sports Traumatol Arthrosc. 2024 Nov 4;33(5):1844–52. doi: 10.1002/ksa.12531 (PMC12022830; doi:10.1002/ksa.12531)
Supplement: Supplementary file 3 — Supporting information. [file KSA-33-1844-s001.docx]

**Appendix 3:** QUADAS-2 checklist

*QUADAS-2 Bias:*

|  | **PATIENT SELECTION** | **INDEX TEST** | **REFERENCE STANDARD** | **FLOW AND TIMING** |
| --- | --- | --- | --- | --- |
| Barrow 2022 | Low | Low | Unclear | High |
| Chen 2021 | Low | Low | Unclear | High |
| Godinho 2021 | High | Low | Low | Low |
| Gyftopoulos 2015 | Low | Low | Unclear | High |
| Yang 2018 | Low | Low | Unclear | High |
| Verweij 2023 | High | Low | Unclear | High |

*QUADAS-2 Applicability:*

|  | **PATIENT SELECTION** | **INDEX TEST** | **REFERENCE STANDARD** |
| --- | --- | --- | --- |
| Barrow 2022 | Low | Low | Unclear |
| Chen 2021 | Low | Low | Unclear |
| Godinho 2021 | High | Low | Low |
| Gyftopoulos 2015 | Low | Low | Unclear |
| Yang 2018 | Low | Low | Unclear |
| Verweij 2023 | Low | Low | Unclear |
